# microRNA expression profiling on individual breast cancer patients identifies novel panel of circulating microRNA for early detection

**DOI:** 10.1038/srep25997

**Published:** 2016-05-16

**Authors:** Rimi Hamam, Arwa M. Ali, Khalid A. Alsaleh, Moustapha Kassem, Musaed Alfayez, Abdullah Aldahmash, Nehad M. Alajez

**Affiliations:** 1 Stem Cell Unit, Department of Anatomy, College of Medicine, King Saud University, Riyadh 11461, Kingdom of Saudi Arabia; 2 Medical oncology unit, Department of Medicine, King Saud University, Riyadh 11461, Kingdom of Saudi Arabia; 3 Medical Oncology Department, South Egypt Cancer Institute, Assuit University, Egypt; 4 KMEB, Department of Endocrinology, University of Southern Denmark, Odense, Denmark; 5 Prince Naif Health Research Center, King Saud University, Riyadh 11461, Kingdom of Saudi Arabia

## Abstract

Breast cancer (BC) is the most common cancer type and the second cause of cancer-related death among women. Therefore, better understanding of breast cancer tumor biology and the identification of novel biomarkers is essential for the early diagnosis and for better disease stratification and management choices. Herein we developed a novel approach which relies on the isolation of circulating microRNAs through an enrichment step using speed-vacuum concentration which resulted in 5-fold increase in microRNA abundance. Global miRNA microarray expression profiling performed on individual samples from 23 BC and 9 normals identified 18 up-regulated miRNAs in BC patients (p(corr) < 0.05). Nine miRNAs (hsa-miR-4270, hsa-miR-1225-5p, hsa-miR-188-5p, hsa-miR-1202, hsa-miR-4281, hsa-miR-1207-5p, hsa-miR-642b-3p, hsa-miR-1290, and hsa-miR-3141) were subsequently validated using qRT-PCR in a cohort of 46 BC and 14 controls. The expression of those microRNAs was overall higher in patients with stage I, II, and III, compared to stage IV, with potential utilization for early detection. The expression of this microRNA panel was slightly higher in the HER2 and TN compared to patients with luminal subtype. Therefore, we developed a novel approach which led to the identification of a novel microRNA panel which was upregulated in BC patients with potential utilization in disease diagnosis and stratification.

Despite recent advances in cancer management and therapy, cancer remains the second leading cause of death worldwide. Breast cancer (BC) is the most common cancer type and the second cause of cancer-related mortality among women. Annually, the number of women who are newly diagnosed with breast cancer is going to exceed 235,000 and there are around 40,000 deaths as a result of breast cancer in the United States^1^. In the Kingdom of Saudi Arabia, breast cancer is the ninth leading cause of death among women in 2010^2^. Furthermore, breast cancer constitutes around 25% of all new registered cancer incidence among Saudi women whereas 1,308 new breast cancer cases were reported during the year 2009^2^. The incidence rate of breast cancer in Saudi Arabia is also expected to increase over the next decades as a result of the population’s growth and aging.

Better understanding of breast cancer tumor biology and the identification of novel biomarkers is essential for the early diagnosis and for better disease stratification and management choices. During the recent years, microRNA (miRNA) has become increasingly recognized as important regulator of both normal and cancer cell biology^3^,^4^,^5^. Global profiling of cancer tissue versus normal tissue has identified several dysregulated miRNAs in different human cancers^6^,^7^,^8^. However, this approach requires invasive procedures such as biopsy or surgical intervention in order to acquire the representative tissue to be analyzed. Therefore, finding a non-invasive approach for early diagnosis and management of different human disease, such as cancer, has always been challenging task. Several studies have reported the potential utilization of circulating biomarkers in different body fluids, such as serum and plasma, as diagnostic and prognostic tools for different types of cancers, while some studies have reported the use of circulating miRNA for cancer detection and prognostic stratification. However, one limitation of the previous studies is that these studies most often examined the expression of defined set of miRNAs or pooled samples from different patients and then assessed miRNA expression using qRT-PCR or other methods, which limits the novelty of the identified miRNA as well as the inability to perform individual data analysis as related to disease condition^9^,^10^,^11^,^12^. In current study, we established a novel and unbiased approach where we isolated circulating miRNAs from breast cancer patients, we employed speed-vacuum centrifugation step to increase the abundance of miRNAs and subsequently conducted global miRNA profiling on individual patient specimens. Our approach led to the identification of a novel 18-microRNA panel which was upregulated in breast cancer patients as early as Stage I and Stage II.

## Results

### Distinct microRNA expression profile in patients with breast cancer compared to healthy controls

**Figure 1:**
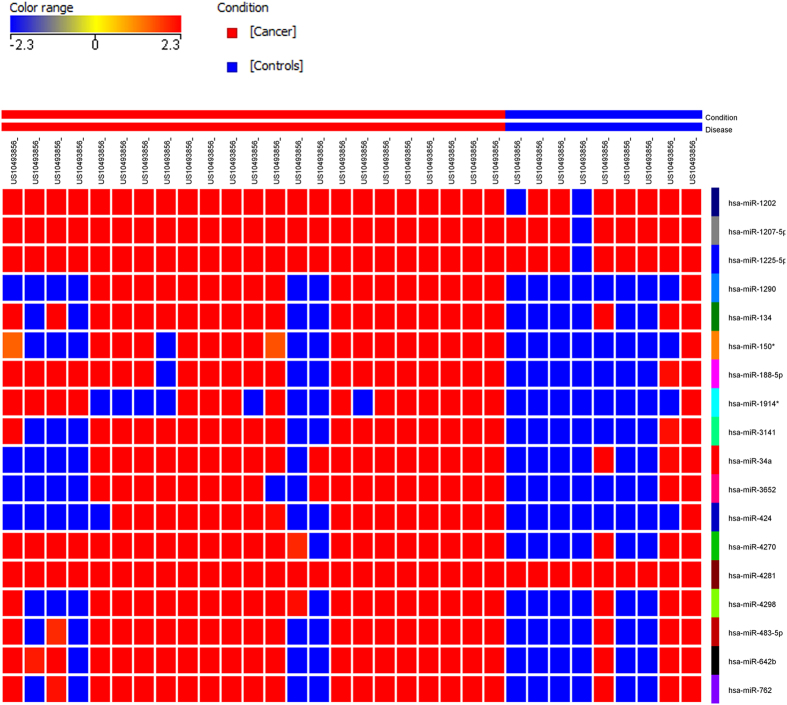
Heatmap depicting the expression of 18 circulating miRNAs in patients with breast cancer (BC) compared to normal healthy controls. Heatmap of individual plasma samples of 9 control and 23 patients with BC. The miRNA expression levels exhibited ≥2.0 fold changes and p ≤ 0.05 are presented. Each column represents an individual sample and each row represents a single miRNA. Expression level of each miRNA in a single sample is depicted according to the color scale.

**Figure 2:**
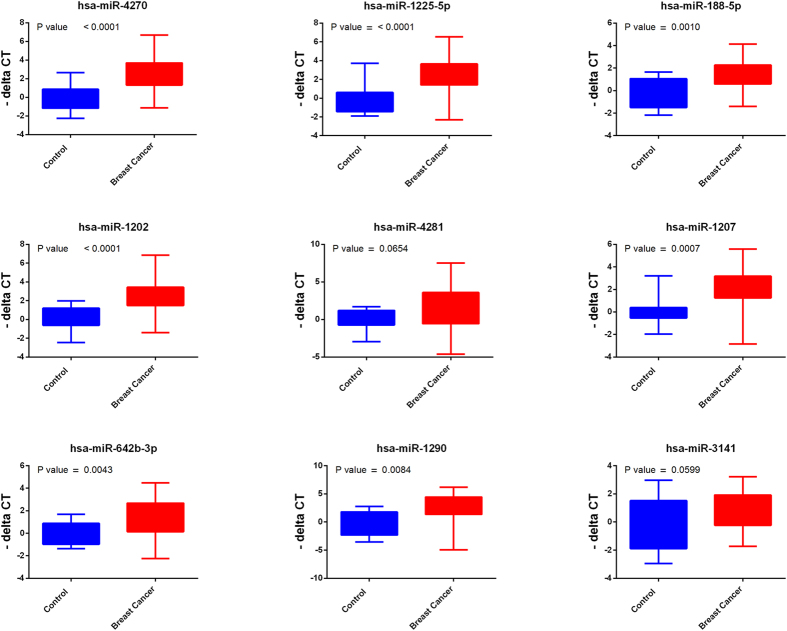
Validation of the expression of 9 miRNAs identified from microarray data employing plasma or serum from patients with breast cancer (BC, n = 46) and healthy controls (n = 14) using quantitative Real-Time PCR. P values were calculated using unpaired t-test and are indicated on each plot. Data are presented as “−delta CT” using box and whiskers plots.

**Figure 3:**
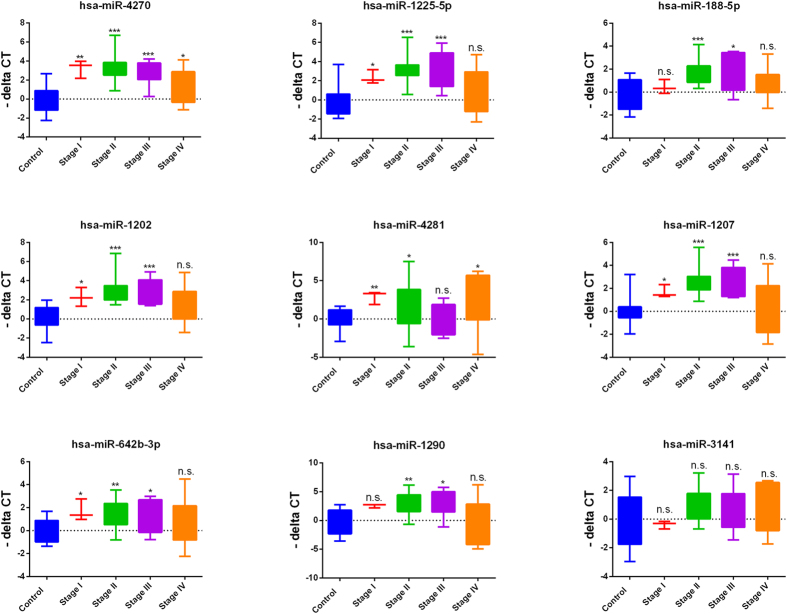
Expression of 9 circulating miRNAs according to breast cancer stage. The expression of a group of 9 circulating miRNAs measured using qRT-PCR in normal (n = 14) or patients with breast cancer (BC, n = 46) are plotted as a function of cancer stage. P values were calculated using unpaired t-test. Data are presented as “−delta CT” using box and whiskers plots. *p < 0.05, **p < 0.005, ***p < 0.0005, ****p < 0.00005, n.s., not significant.

**Figure 4:**
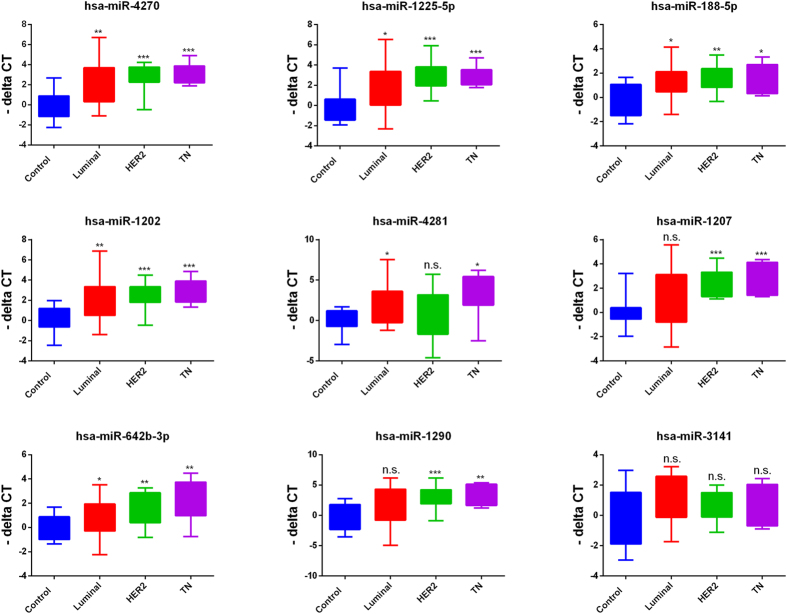
Expression of 9 circulating miRNAs according to breast cancer molecular subtype. The expression of a group of 9 circulating miRNAs measured using qRT-PCR in normal (n = 14) or patients with breast cancer (BC, n = 46) are plotted as a function of BC molecular type. P values were calculated using unpaired t-test. Data are presented as “−delta CT” using box and whiskers plots. *p < 0.05, **p < 0.005, ***p < 0.0005, ****p < 0.00005, n.s., not significant, TN: triple negative, HER2: HER2+.

Circulating RNAs were isolated using Norgen’s slurry format kit. To further increase the concentration of isolated RNA, we utilized speed-vacuum concentration approach. As shown in supplementary Fig. 1, this approach increased the detection limit for circulating miRNA in these samples ~4.6 fold. Circulating miRNAs were identified by performing global miRNA microarray expression profiling on 23 breast cancer and 9 normal control samples. Using Benjamini-Hochberg False Discovery Rate (FDR) multiple testing correction method (p(corr) < 0.05) and two-fold change cut-off, we identified 18 up-regulated miRNAs in samples obtained from patients with breast cancer compared to healthy controls. Individual data are presented in the heatmap in Fig. 1. Breast cancer-associated miRNAs identified were: hsa-miR-4270, hsa-miR-1225-5p, hsa-miR-188-5p, hsa-miR-1202, hsa-miR-4281, hsa-miR-1207-5p, hsa-miR-642b-3p, hsa-miR-1290, hsa-miR-3141, hsa-miR-150-3p, hsa-miR-4298, hsa-miR-483-5p, hsa-miR-134, hsa-miR-762, hsa-miR-1914-3p, hsa-miR-34a-5p, hsa-miR-3652, and hsa-miR-424-5p (Table 1). Among the identified miRNAs, hsa-miR-4270 was the most significantly upregulated miRNA in patients’ samples compared to controls (p value = 3.5 × 10^−6^, p value (Corr) = 0.001). The top nine miRNAs (based on p value, Table 1) : hsa-miR-4270, hsa-miR-1225-5p, hsa-miR-188-5p, hsa-miR-1202, hsa-miR-4281, hsa-miR-1207-5p, hsa-miR-642b-3p, hsa-miR-1290, and hsa-miR-3141 were subsequently chosen for further validation in a cohort of 46 BC and 14 normal controls, including 23 BC and 8 normals which were used for the microarray profiling. Data presented in Fig. 2 collectively validated the microarray data. Interestingly, the expression of the nine miRNAs was slightly higher in Stage I, Stage II, and Stage III compared to Stage IV (Fig. 3). Anova analysis revealed significant difference in the expression of hsa-miR-4270, hsa-miR-1225-5p, hsa-miR-1202, hsa-miR-1207, and hsa-miR-1290 as function of cancer stage. When the expression of those nine miRNAs was plotted as function of breast cancer molecular subtype, similar trends of miRNA expression were overall seen, whereas the expression was slightly higher in the HER2 and TN compared to the luminal cancer subtype. Additionally, patients with the luminal subtype exhibited the largest degree of heterogeneity, possibly due to further luminal A/B sub classification (Fig. 4).

## Discussion

Identifying reliable blood biomarkers for early diagnosis or prognostic stratification of various human diseases is an area of intensive investigation. Proteins, DNA, and mRNA could be detected in the circulation of cancer patients and have been suggested in some studies to reflect disease activity^13^,^14^,^15^. miRNAs have recently emerged as providing reliable biomarkers for disease status in a number of cancer patients as well as in other diseases, due to their stability and ease of detection^10^,^16^,^17^. Current protocols employed to detect circulating miRNAs rely mostly on using either a PCR-based method for measuring the expression of selected panel of miRNAs or the utilization of pooled serum/plasma samples and running a more robust assay such as miRNA microarrays. However, these approaches in general do not lead to the identification of novel miRNA or studying their relevance at the individual patient’s level. In our studies we have employed individual patients’ and control’s samples and utilized an unbiased discovery approach using miRNA microarray profiling.

Several of the established protocols for miRNA isolation from serum and plasma require a large sample volume to obtain sufficient RNA for down-stream analysis. Thus, clinical serum and plasma samples from several patients are frequently pooled^10^,^18^, which may compromise statistical analyses by preventing using the individual patient as the statistical unit, that may lead to erroneous conclusions. In our study, we have avoided this limitation as we developed a novel approach which relies on the isolation of circulating microRNAs through an enrichment step using speed-vacuum concentration that resulted in ~5-fold enrichment. In addition, we employed an unbiased discovery approach through using microarray chips which can detect more than 1700 human-specific miRNAs.

We identified 18 novel miRNAs, which were upregulated in BC patients compared to matched healthy controls. The expression of nine selected miRNAs was subsequently validated in a cohort of 46 breast cancer and 14 normal subjects, which collectively corroborated the microarray data.

Some of the identified miRNAs have previously been reported to be associated with a number of human diseases, hsa-miR-134 is upregulated sera of patients with acute myocardial infarction^19^ while hsa-miR-483-5p levels were increased in sera of patients with adrenocortical tumors and pulmonary tuberculosis^20^,^21^. Elevated expression of hsa-miR-1290 was reported in prostate cancer and in fatty liver disease^22^,^23^. To our knowledge, none of the other miRNAs identified in our study has been reported to be enriched in the circulation of other human diseases. Interestingly, those miRNAs were also detected in the circulation of BC patients with early stage (I and II), which suggest potential utilization of this miRNA panel for early detection.

Some miRNAs which have been reported to be enriched in breast cancer tissues, such as hsa-miR-21 and hsa-miR-155, were not significantly upregulated in the circulation of breast cancer patients in our study. Therefore our data suggest that circulating microRNAs are unlikely to be derived from the tumor itself, but rather reflect the generalized homeostatic responses during health and disease. In support of this hypothesis, pathway analysis on the predicted gene targets for the identified miRNAs revealed potential role in important cellular processes (supplementary Fig. 2). Also, hsa-miR-1202 has recently been reported to be down-regulated in patients with depression^24^ and thus mental state may contribute to some changes in circulating miRNAs profile. In addition, we observed upregulated expression of two HSV2 miRNAs in the circulation of breast cancer patients (supplementary Fig. 3).

In conclusion, we have identified a novel panel consisting of 18 circulating miRNAs that is enriched in patients with breast cancer and their presence was higher in patients with stage I, II, and III suggesting it possible utilization for early diagnosis and disease stratification. Similar strategy and methodology can also be utilized for detecting circulating miRNAs in other human diseases.

## Materials and Methods

### Ethics statement

The clinical study and blood collection were approved by Institutional Research Ethics Board at the King Saud University College of Medicine (Riyadh, Riyadh, Saudi Arabia). The methods were carried out in accordance with the approved guidelines. Informed consent was obtained from all subjects.

### Patient and blood collection

Blood samples from 46 breast cancer patients were obtained from patients undergoing standard treatment at the King Khaled University Hospital (Riyadh, Saudi Arabia). Controls samples were obtained from 14 healthy women. The clinical information of subjects included in current study and their tumor characteristics are listed in Table 2. Samples with signs of hemolysis were excluded from the study.

### Plasma and serum preparation and total RNA isolation

Plasma and serum were prepared from freshly collected whole blood samples (EDTA-coated tubes for plasma collection or regular tubes for serum collection) by centrifugation at 2,000 rpm for 15 minutes at room temperature. Collected plasma and serum were frozen at −80 °C. Circulating RNAs were extracted from ~3.5 mL of the collected plasma or serum using Norgen’s Plasma/Serum Circulating and Exosomal RNA Purification Kit Slurry Format (Norgen Biotek, Thorold, Ontario, Canada) according to the manufacturer’s protocol, followed by RNA elution in 100 *μ*l of elution buffer. Twenty five *μ*l of the purified total RNA were dried by speed-vacuum centrifugation (60 minutes at RT), and were then reconstituted in five *μ*l of DNAse and RNAse-free water (5-fold enrichment). Normalization across samples was done by using equal volume of input plasma or serum (~3.5 ml) for miRNA isolation from each sample. Isolated miRNA fraction was subsequently used for global miRNA profiling by microarray (after drying and reconstitution) or by qRT-PCR (without drying and reconstitution).

### miRNA expression profiling

miRNA expression profiling was conducted on the isolated miRNA fraction from 23 patients with breast cancer and nine normals. Two microliter of the extracted RNA (after 5-fold concentration) was labeled and subsequently hybridized to the Agilent Human SurePrint G3 8 × 60 k v21 miRNA microarray chip as described before^6^. Subsequently, data were imported into GeneSpring 13.0 software for analysis (Agilent Technologies). Differentially expressed miRNAs in cancer patients *versus* healthy control samples were determined using a corrected p-value (≤0.05, Benjamini-Hochberg multiple testing correction method). MicroRNAs showing ≥2.0 fold change were considered significant.

### miRNA validation by qRT-PCR

Validation of selected number of miRNA was carried out on circulating miRNA fraction isolated from either plasma or serum samples from 46 BC and 14 controls. Validation was conducted using the miProfile™ Custom miRNA qPCR Array (GeneCopoeia, Rockville, MD, United States). For miRNA expression detection, five μl of the purified total RNA were used as input according to the manufacturer’s protocol. All primers used in the current study were provided by GeneCopoeia, except for hsa-miR-21 primers which were obtained from Applied Biosystems. The relative expression levels were expressed as “−delta CT” of breast cancer patients compared to normal controls. Quantification of hsa-miR-21 in plasma samples before and after speed vacuum concentration was conducted using Taqman microRNA assay kit (Applied Biosystems, USA) as described before^5^.

#### Molecular profiling of breast cancer patients

Molecular profiling of BC patients was conducted in accordance with previously published criteria and based on the pathological characteristics of patients utilized in current study^25^,^26^.

### Statistical analysis

Statistical analyses and graphing were conducted using GraphPad Prism 6.0 software (GraphPad, San Diego, CA, USA). *P*-values were calculated using the two-tailed *t*-test or one way anova analysis.

## Additional Information

**How to cite this article**: Hamam, R. *et al*. microRNA expression profiling on individual breast cancer patients identifies novel panel of circulating microRNA for early detection. *Sci. Rep.* **6**, 25997; doi: 10.1038/srep25997 (2016).

## Supplementary Material

Supplementary Information

## Figures and Tables

**Table 1:** Differentially expressed circulating plasma miRNAs in 23 breast cancer patients compared to 9 normal controls.

Updated_systematic_name	mirbase accession No	p (Corr)	p	Regulation	FC
hsa-miR-4270	MIMAT0016900	0.001193408	5.35E-06	up	182.9
hsa-miR-1225-5p	MIMAT0005572	0.004337585	7.78E-05	up	22.7
hsa-miR-188-5p	MIMAT0000457	0.004337585	6.59E-05	up	73.0
hsa-miR-1202	MIMAT0005865	0.006776397	1.52E-04	up	27.3
hsa-miR-4281	MIMAT0016907	0.019025994	5.12E-04	up	11.6
hsa-miR-1207-5p	MIMAT0005871	0.0202241	7.26E-04	up	14.7
hsa-miR-642b-3p	MIMAT0018444	0.0202241	6.99E-04	up	62.9
hsa-miR-1290	MIMAT0005880	0.022832992	9.22E-04	up	45.9
hsa-miR-3141	MIMAT0015010	0.029752607	0.001334	up	50.7
hsa-miR-150-3p	MIMAT0004610	0.033017445	0.001629	up	43.7
hsa-miR-4298	MIMAT0016852	0.03568337	0.00192	up	40.2
hsa-miR-483-5p	MIMAT0004761	0.038129777	0.002223	up	42.3
hsa-miR-134	MIMAT0000447	0.04201405	0.002826	up	42.2
hsa-miR-762	MIMAT0010313	0.04201405	0.0028	up	43.1
hsa-miR-1914-3p	MIMAT0007890	0.044369638	0.003895	up	30.8
hsa-miR-34a-5p	MIMAT0000255	0.044369638	0.004159	up	29.5
hsa-miR-3652	MIMAT0018072	0.044369638	0.003801	up	44.3
hsa-miR-424-5p	MIMAT0001341	0.044369638	0.003333	up	43.0

**Table 2:** clinical information of patients included in current study and their tumor characteristics and normal control.

Cancer	N = 46	%
Age, y		
Median age	50 y	
Range	27–71 y	
Gender		
Female	46	100%
Stage		
I	3	6.5%
II	17	37.0%
III	10	21.7%
IV	13	28.3%
NA	3	6.5%
Molecular Subtype		
Luminal	22	48%
HER2	17	37%
TN	7	15%
Recurrence		
Recurrent	4	8.6%
Non Recurrent	42	91.4%
Normal	N = 14	
Age, y		
Median age	36.5 y	
Range	26–60 y	

HER2: HER2+, TN: Triple Negative.
