# Supplementary material for: microRNA expression profiling on individual breast cancer patients identifies novel panel of circulating microRNA for early detection
Source: Sci Rep. 2016 May 16;6:25997. doi: 10.1038/srep25997 (PMC4867432; doi:10.1038/srep25997)
Supplement: Supplementary Information [file srep25997-s1.pdf]

microRNA expression profiling on individual breast cancer patients identifies novel panel of circulating microRNA for early detection

Rimi Hamam[1], Arwa M Ali[2,3], Khalid A Alsaleh[2], Moustapha Kassem[1,4], Musaed Alfayez[1], Abdullah Aldahmash[1,5], Nehad M Alajez[1]*

[1]Stem Cell Unit, Department of Anatomy, College of Medicine, King Saud University, Riyadh 11461, Kingdom of Saudi Arabia
[2]Medical oncology unit, Department of Medicine, King Saud University, Riyadh 11461, Kingdom of Saudi Arabia
[3]Medical Oncology Department, South Egypt Cancer Institute, Assuit University, Egypt
[4]KMEB, Department of Endocrinology, University of Southern Denmark, Odense, Denmark
[5]Prince Naif Health Research Center, King Saud University, Riyadh 11461, Kingdom of Saudi Arabia

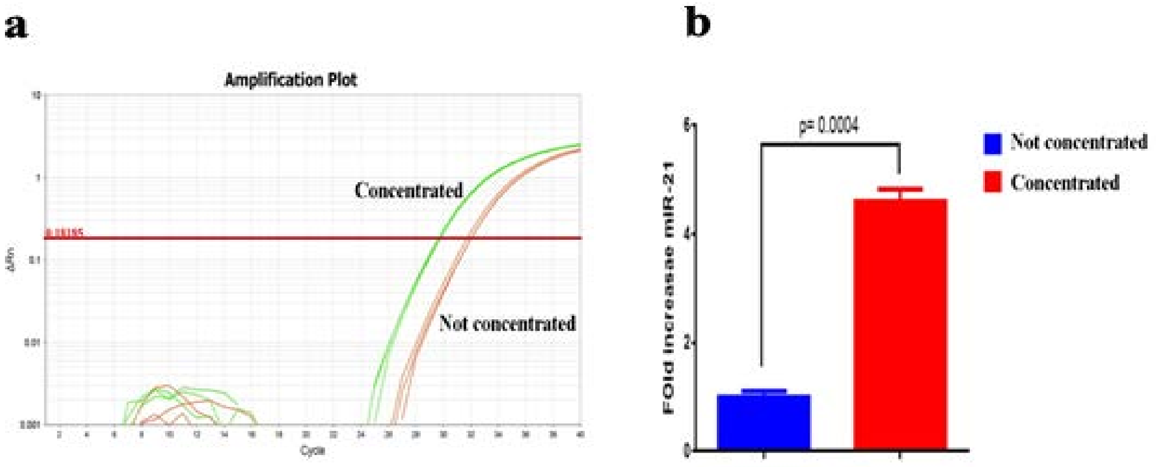

Supplementary figure 1. Isolation and concentration of circulating miRNAs from breast cancer patient' plasma. **(a)** Expression of miR-21 in representative breast cancer patient before (green) and after (red) speed-vacuum concentration and quantification using quantitative Real-Time PCR. **(b)** Data from (a) are presented as mean +/- SD, n=3.

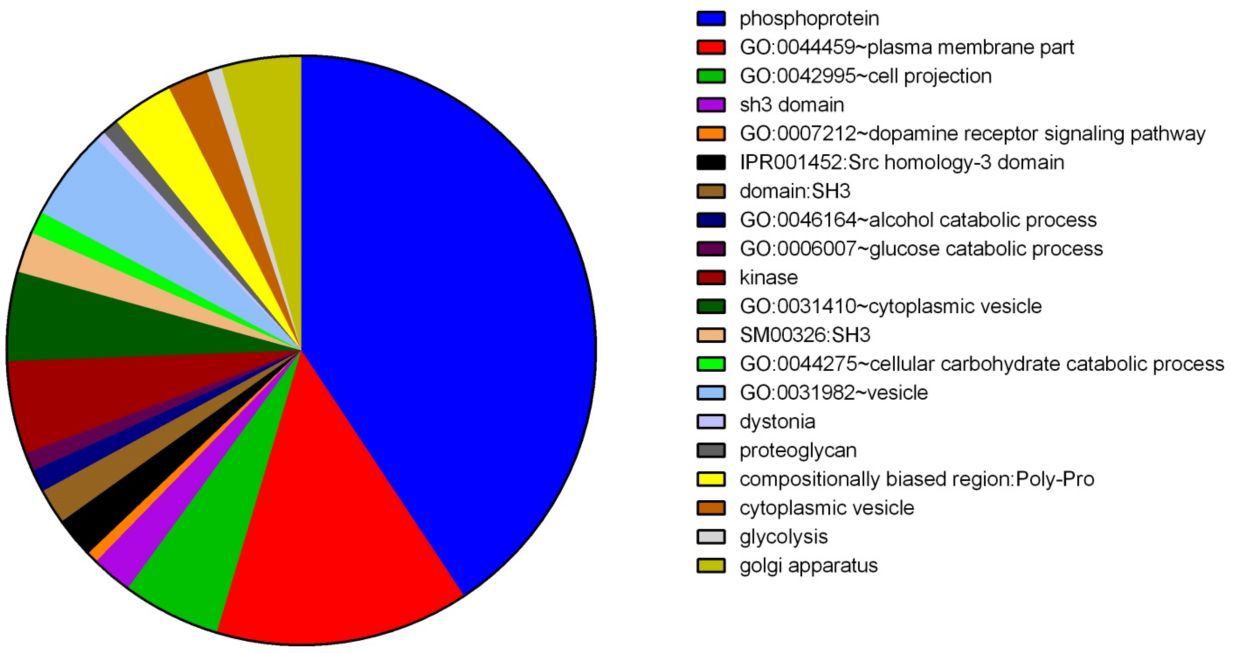

Supplementary figure 2. Pie chart illustrating the distribution of the top 20 pathway designations for the predicted gene targets for 18 upregulated miRNAs in breast cancer circulation based on TargetScan database. The pie size corresponds to the number of matched entities.

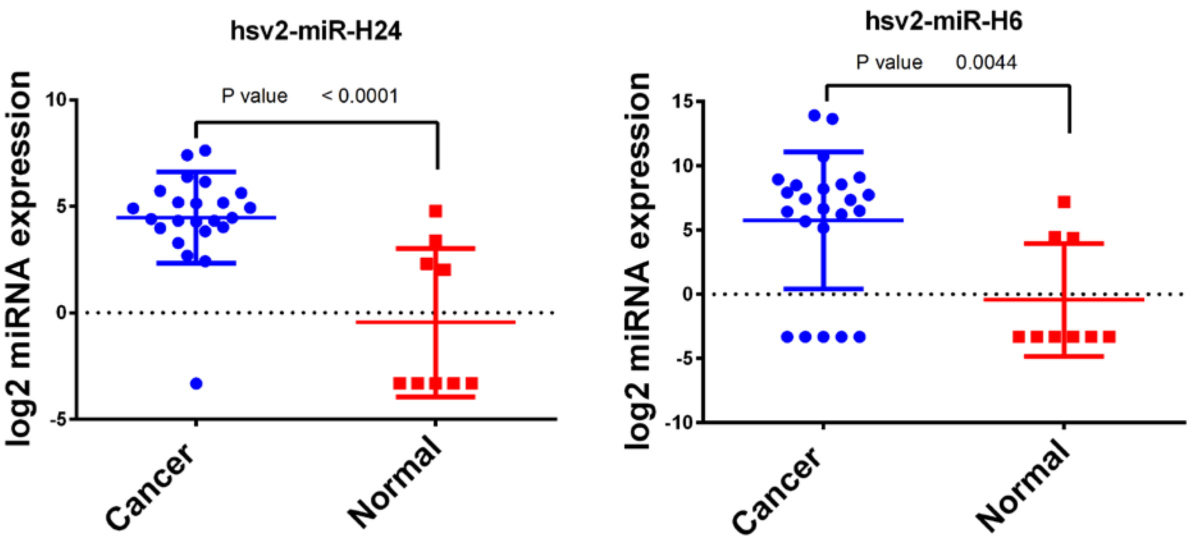

Supplementary figure 3. Expression of hsv2-miR-H24 and hsv2-miR-H6 in the circulation of breast cancer patients (n=23) and normal controls (n=9) based on microRNA microarray expression. Data are presented as log2 expression. Two tailed t-test was used to calculate significance.
